# Non-local terahertz photoconductivity in the topological phase of Hg_1−*x*_Cd_*x*_Te

**DOI:** 10.1038/s41598-021-81099-6

**Published:** 2021-01-15

**Authors:** A. S. Kazakov, A. V. Galeeva, A. I. Artamkin, A. V. Ikonnikov, L. I. Ryabova, S. A. Dvoretsky, N. N. Mikhailov, M. I. Bannikov, S. N. Danilov, D. R. Khokhlov

**Affiliations:** 1 Physics Department, M.V. Lomonosov Moscow State University, Moscow, 119991 Russia; 2 Chemistry Department, M.V. Lomonosov Moscow State University, Moscow, 119991 Russia; 3 A.V. Rzhanov Institute of Semiconductors Physics, Siberian Branch of RAS, Novosibirsk, 630090 Russia; 4 P.N. Lebedev, Physical Institute of RAS, Moscow, 119991 Russia; 5 Faculty of Physics, University of Regensburg, 93053 Regensburg, Germany

**Keywords:** Topological insulators, Topological insulators, Terahertz optics, Two-dimensional materials

## Abstract

We report on observation of strong non-local photoconducitivity induced by terahertz laser pulses in non-zero magnetic field in heterostructures based on Hg_1−*x*_Cd_*x*_Te films being in the topological phase. While the zero-field non-local photoconductivity is negligible, it is strongly enhanced in magnetic fields ~ 0.05 T resulting in appearance of an edge photocurrent that exceeds the respective dark signal by orders of magnitude. This photocurrent is chiral, and the chirality changes every time the magnetic field or the electric bias is reversed. Appearance of the non-local terahertz photoconductivity is attributed to features of the interface between the topological film and the trivial buffer.

## Introduction

Studies of topologically non-trivial materials, such as 2D- and 3D topological insulators, topological crystalline insulators and others—are among the hottest topics in the modern solid state physics. Due to the strong spin–orbit interaction, the conduction and valence bands in the bulk are inverted, and high-mobility topological electronic states with the Dirac dispersion relation and the spin direction locked perpendicular to the momentum direction necessarily appear at the material surface^1^. Existence of the topological surface electron states in topological insulators was first predicted theoretically^2,3^ and then observed experimentally through the ARPES measurements^4–7^. These experiments however do not give information on the electron transport along the topological surface electron states. On the other hand, direct transport measurements meet substantial difficulties due to high conductivity in the bulk of most of the topologically non-trivial materials that shunts the conductivity via the topological surface states. Extraction of the contribution of topological electron states to the electron transport is a sophisticated task that is not always unambiguous^1^.

A promising experimental approach that allows avoiding these problems is the optoelectronic probing of surface electron states^8–10^. In many cases, the photoelectric effects are not sensitive to the bulk conductivity, and it turned out to be possible to access features of the topological surface electron states through photogalvanic measurements^11–14^.

One of the key distinguishing features of the electronic transport in the 2D topological insulators is the non-local edge conductivity^15–17^. These measurements require a special mesa design when the bulk current flows only in a restricted local area of the sample, and the voltage drop is measured at a remote contact couple at the sample edge. The non-local conductivity was observed experimentally in HgTe quantum wells^15^ and other semiconductor structures^18,19^.

In this study, we combine the two experimental approaches mentioned above, for a set of heterostructures based on thick Hg_1-*x*_Cd_*x*_Te films. We directly demonstrate appearance of the edge photocurrents upon terahertz photoexcitation in heterostructures based on topological Hg_1-*x*_Cd_*x*_Te films. These photocurrents exceed the equilibrium values by orders of magnitude. The effect is absent in the trivial phase-based films.

## Results

Hg_1-*x*_Cd_*x*_Te solid solutions reveal the inverted respective positions of the conduction and light valence bands providing appearance of topological surface electron states at *x* < 0.16. Instead, the trivial band ordering occurs at *x* > 0.16, so a composition-driven topological phase transition is observed^20–24^. In the vicinity of this phase transition, the characteristic energy spectrum values are on the order of dozens of meV which corresponds to the terahertz spectral range. Modern techniques of the epitaxial growth allow synthesizing Hg_1−*x*_Cd_*x*_Te films with low free carrier concentration ~ 10^14^ cm^−3^^25,26^. It means that excitation of the sample electron system with the terahertz radiation may change considerably the equilibrium carrier concentration and/or mobility providing appearance of the photoconductivity.

**Figure 1:**
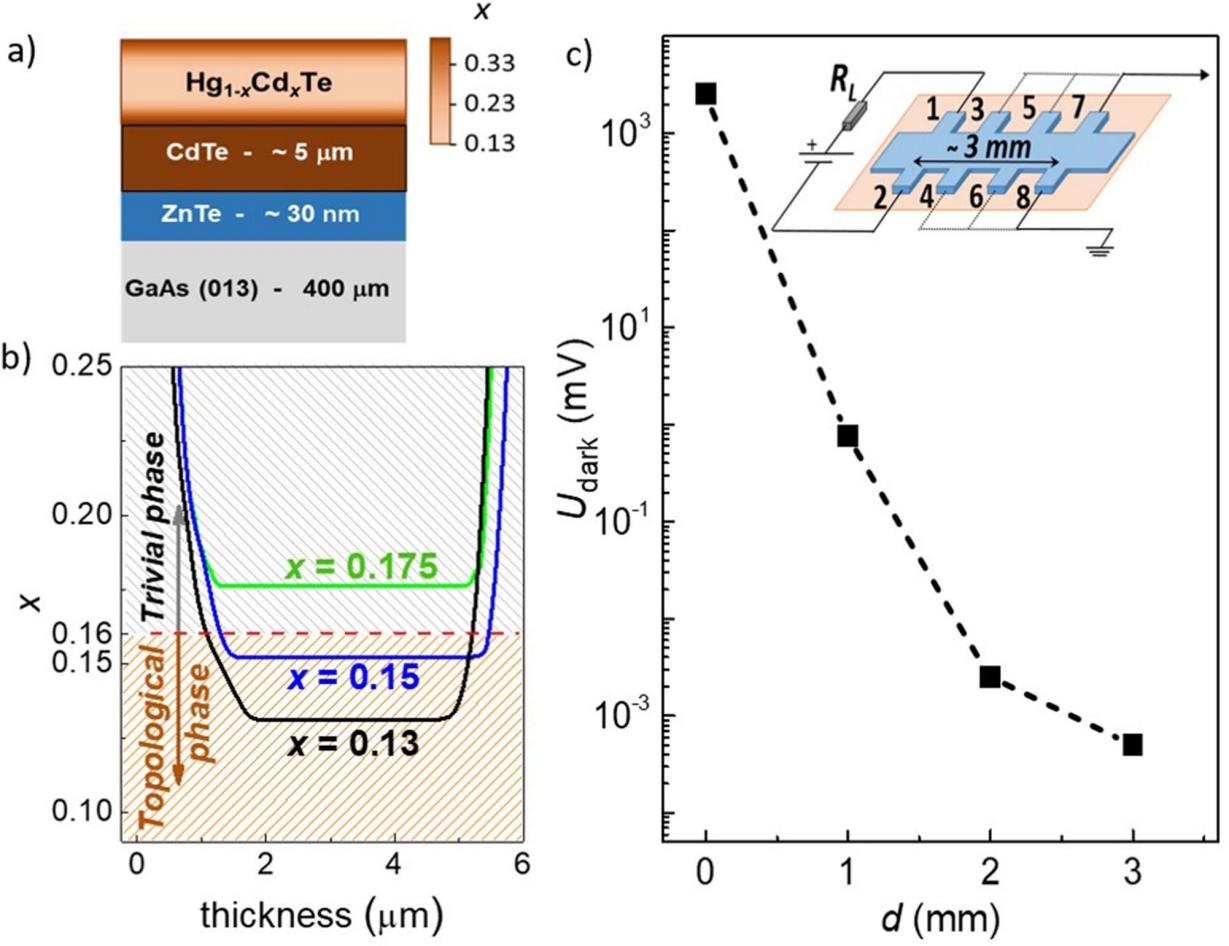
Details of the sample characterization. **(a)** Sequence of layers in heterostructures under study. **(b)** CdTe content *x* distribution in the active parts of heterostructures. **(c)** The potential drop as a function of the distance *d* between the current leads and the potential probes along the sample in the absence of external illumination. The inset shows a typical geometry of the non-local conductivity experiments.

In the non-local geometry of measurements, the external bias is applied to a couple of adjacent contacts while the potential drop is taken at a remote contact pair. An example of the respective experimental geometry is shown in the inset to the Fig. 1c. The DC external bias is applied to the contacts 1–2, and the voltage is measured across the potential probe couples 3–4, 5–6 or 7–8. In the absence of external illumination, this potential drop decreases exponentially as a function of the distance *d* between the current leads and the potential probes. This dependence is presented in the Fig. 1c. Please note that the datapoint at *d* = 0 corresponds to the 2-probe measurements, while other points are taken using the 4-probe technique. The potential drop does not exceed 800 µV even for the contact couple 3–4 which is closest to the current leads, while it is below 1 µV (instrumental zero) for the most remote contact couple 7–8. Such an exponential dependence is typical for currents flowing via the sample bulk.

The terahertz photoconductivity measurements were performed at *T* = 4.2 K in the Faraday geometry at magnetic fields *B* < 1 T. Let us denote the magnetic field *B* direction coinciding with the incident radiation flux direction as the positive one *B*^+^, and the opposite direction as negative *B*^*-*^.

**Figure 2:**
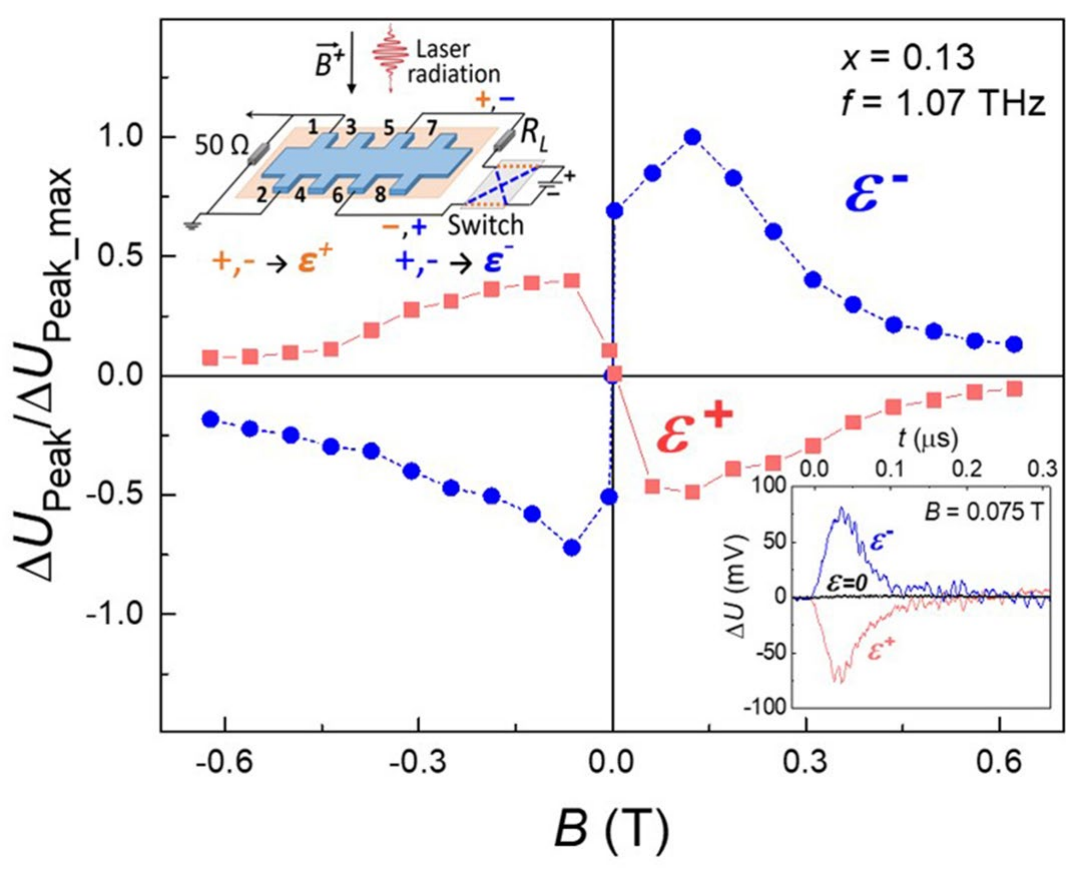
Magnetic field dependence of the photoresponse. The magnetic field dependence of the photosignal amplitude normalized to its maximal value taken using the configuration shown in the top inset. In the bottom inset: photocurrent kinetics taken at the zero bias, as well as for the two opposite electric bias polarities. *ε*^+^ denotes the bias polarity marked by red “ + ” and “−”, while *ε*^*-*^ implies the bias polarity marked by blue “ + ” and “−”.

In the zero magnetic field, the non-local photocurrent signal is practically absent. As the magnetic field grows, the non-local photocurrent appears in the topological phase samples with *x* < 0.16. Typical photocurrent kinetics taken at the zero bias, as well as for two opposite electric bias polarities taken for the remote couple of contacts 1–2 is shown in the bottom inset to the Fig. 2. Two main features of this photosignal must be stressed. First, the photocurrent is zero at the zero bias, and it changes its sign to the opposite one when the bias polarity reverses. Such a behavior is typical for the photoconductivity. Second, the amplitude of the photovoltage drop across the 50 Ω loading resistor is giant: it exceeds by at least 2–3 orders of magnitude the potential drop between the same couple of contacts measured in equilibrium in the absence of terahertz radiation.

The photosignal amplitude ∆*U*_Peak_ field dependence taken at the contact couple 1–2 is presented in the Fig. 2. The effect amplitude grows with increasing field, reaches maximum at ~ 0.08 T, then drops down considerably in higher fields. Surprisingly, the effect changes its sign upon the magnetic field inversion. It means that whereas for the magnetic field direction *B*^*-*^, the photocurrent flows from the positive current lead to the negative one, as it does for the equilibrium current, for the opposite magnetic field direction *B*^+^, the photocurrent flows in the opposite direction—from the negative to the positive current lead.

**Figure 3:**
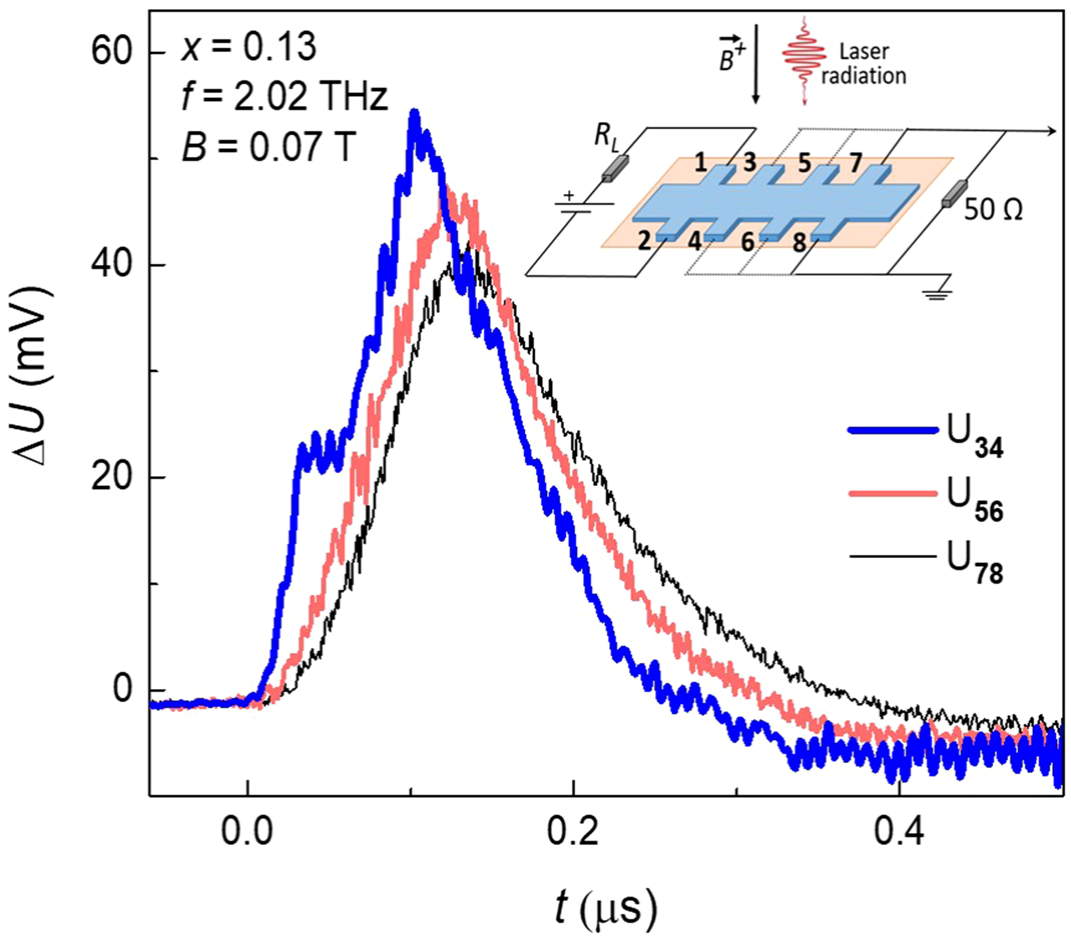
Nonlocal photoresponse probing. Typical photosignal kinetics taken between different couples of the potential leads 3–4, 5–6 and 7–8 while 1–2 are the current leads. The inset shows the experiment geometry. The film composition *x* = 0.13 corresponds to the topological phase, the magnetic field *B*^+^  = 0.07 T, the terahertz laser frequency is 2.02 THz.

Strikingly, the photocurrent signal practically does not depend on the distance between the current leads 1–2 and the remote potential lead couples 3–4, 5–6 and 7–8 (Fig. 3). This is in a drastic contrast with the strong exponential dependence of the potential drop between these contact couples on the distance *d* from the current leads in equilibrium (see Fig. 1).

**Figure 4:**
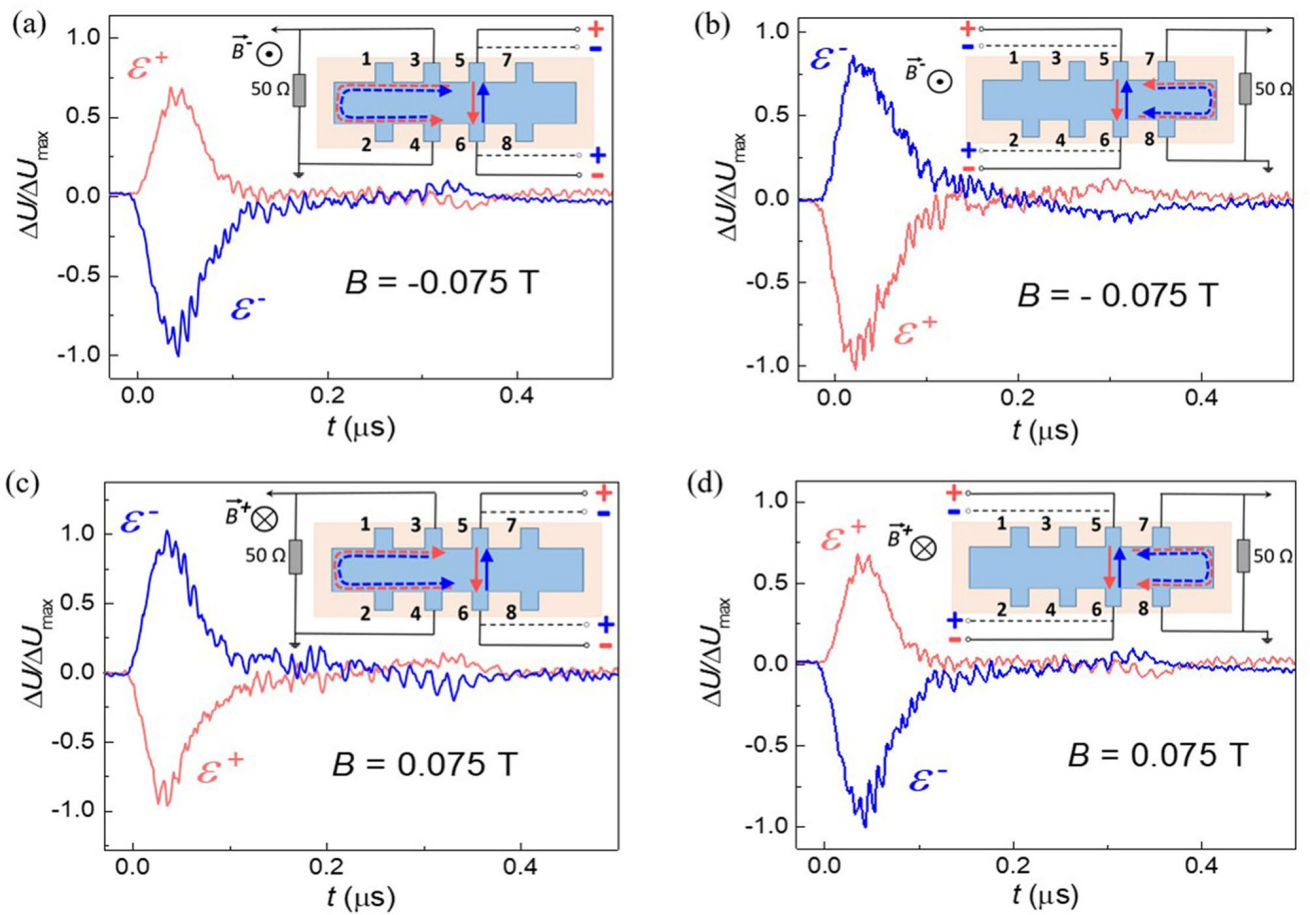
Chirality of the nonlocal photoresponse. The photosignal induced by the laser radiation with the 1.07 THz frequency under external bias applied to the middle couple of contacts 5–6 in the sample with *x* = 0.13. The photosignal measured in the same magnetic field changes its sign when measured to the left **(a)** or to the right **(b)** from the current lead couple 5–6. Changing the magnetic field polarity reverses the photosignal sign at both potential probe couples **(c,d)**. The sketches of the respective experimental geometries are shown in the corresponding insets.

Another astonishing feature of the effect observed is the following. The photocurrent direction depends on the side of a sample with respect to the line connecting the current leads. Let us apply the external bias to the middle couple of contacts 5–6, and measure the photosignal from the adjacent couples 3–4 and 7–8 located at two sides of the line connecting the current leads, in magnetic field. The results are shown in the Fig. 4. The photosignal measured in the same magnetic field, changes its sign when measured to the left (contact couple 3–4) or to the right (contact couple 7–8) from the current lead couple 5–6. Again, changing the magnetic field polarity reverses the photosignal sign at both contact couples. This result means that the non-local photocurrent observed is chiral, i.e. it flows around the sample.

**Figure 5:**
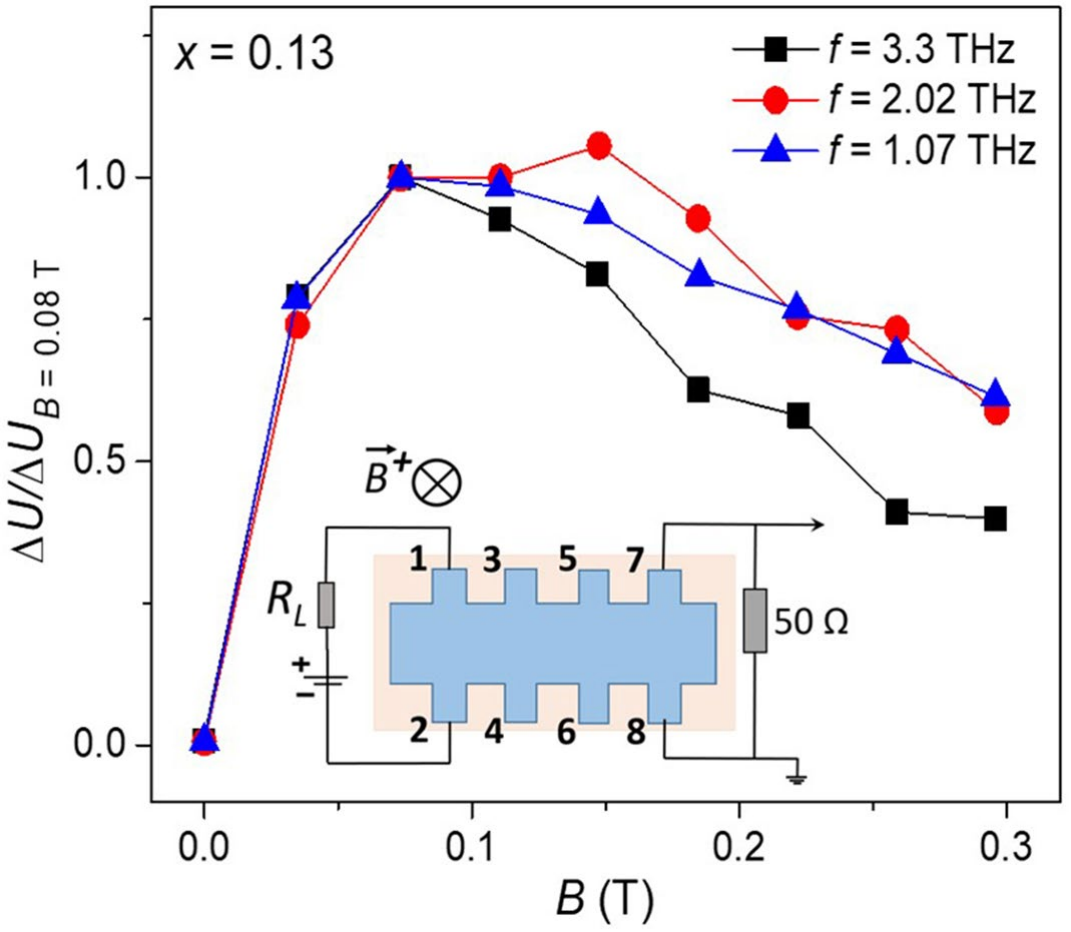
Magnetic field dependence of the effect amplitude taken at different laser frequencies. The effect amplitude is normalized to its value at *B* = 0.08 T. The data are taken for the contact couple (1–2) used as current leads and the contact couple (7–8) as photosignal leads, the magnetic field direction is *B*^+^ (see the inset), the sample composition *x* = 0.13.

The key features of the effect do not depend on the laser radiation frequency from 1.1 to 3.3 THz. Figure 5 presents the magnetic field dependence of the effect amplitude for three different laser frequencies. The data are normalized to the value taken at *B* = 0.08 T. One can see that the magnetic field corresponding to the effect maximum practically does not change for the laser excitation frequencies differing by a factor of 3.

The non-local photoconductivity is not sensitive to the radiation polarization: neither circular, nor linear. This was directly checked by rotating *λ*/2 and *λ*/4 plates introduced into the laser beam.

The effects described above are observed only in films with *x* < 0.16 corresponding to the topological phase. The non-local photoconductivity has not been detected in the film with *x* = 0.175 being in the trivial phase.

## Discussion

Our results show that the terahertz radiation induces very unusual photocurrents in the topological phase films.

First, these photocurrents are flowing via the sample edge, i.e. they are non-local. If they would flow either via the film bulk or surface, their amplitude would drop exponentially upon moving the potential lead couple away from the current leads. The equilibrium potential drop demonstrates this exponential behavior, but not the photocurrent. The edge of the interface between the trivial buffer and the topological film at which 2D topological electron states are expected to form is the most likely candidate for the non-local photocurrent location. Another argument in support of this statement is absence of this kind of photocurrents for the trivial phase film with *x* = 0.175.

Second, the photocurrents change their direction upon inversion of the magnetic field or the bias applied. They are close to zero in the absence of electrical bias or magnetic field.

Third, and most unusual—these photocurrents are chiral, meaning that they flow along the sample edge around a sample. The chirality is reversed every time the applied bias or the magnetic field is reversed.

At the present, there is no satisfactory explanation for all of our observations.

An analogy could come from the comparison of the effect observed with zero-resistance states in the quantum Hall effect, when the current propagates along the 2-D layer edge without dissipation^27^. In the quantum Hall effect, the edge current direction is reversed every time the magnetic field is reversed. These zero-resistive currents are chiral. However, on the contrary to our case, the current chirality depends solely on the magnetic field direction, and not on the polarity of the bias applied.

One more possibility could be related to the terahertz excitation of magnetoplasmons. They also change their chirality upon the magnetic field reversal^28,29^. However, flipping the photocurrent polarity upon bias switching may not be explained by this hypothesis. Besides, a strong THz frequency dependence in the position of the effect maximum in magnetic field is expected for magnetoplasmons, which is not the case (see Fig. 5).

To a certain extent, the chiral photocurrents observed remind motion of an electron under the Lorentz force action along the sample edge. Indeed, the photocurrent changes its chirality every time the magnetic field direction is flipped. Besides, the photocurrent is zero in the zero magnetic field, as the Lorentz force does. The chirality is also changed to the opposite one when the photoelectron velocity along the sample edge is reversed. The latter is obviously induced by the switch of the bias polarity.

It is important to note that for a 2D topological layer, the electron spin direction is linked perpendicularly to its momentum and lies in the same 2D plane, i.e. it is co-directed with the Lorentz force in low magnetic fields. In higher fields, however, the photoelectron spin starts to turn along the magnetic field direction providing appearance of a maximum in the photocurrent magnetic field dependence. Therefore, coincidence of the Lorentz force and photoelectron spin direction seems to be an important factor for the effect appearance.

In general, the chiral photocurrents observed remind a macroscopic orbital motion with the angular momentum directed perpendicularly to the sample plane. Possibly, we observe a consequence of a macroscopic spin–orbit interaction.

On the other hand, it is not clear how is the edge photocurrent chirality chosen, since for the same magnetic field direction, the photocurrent chirality depends on the electric bias polarity. This fact contradicts the apparent symmetry arguments. There should exist a factor that breaks the apparent experiment symmetry.

**Figure 6:**
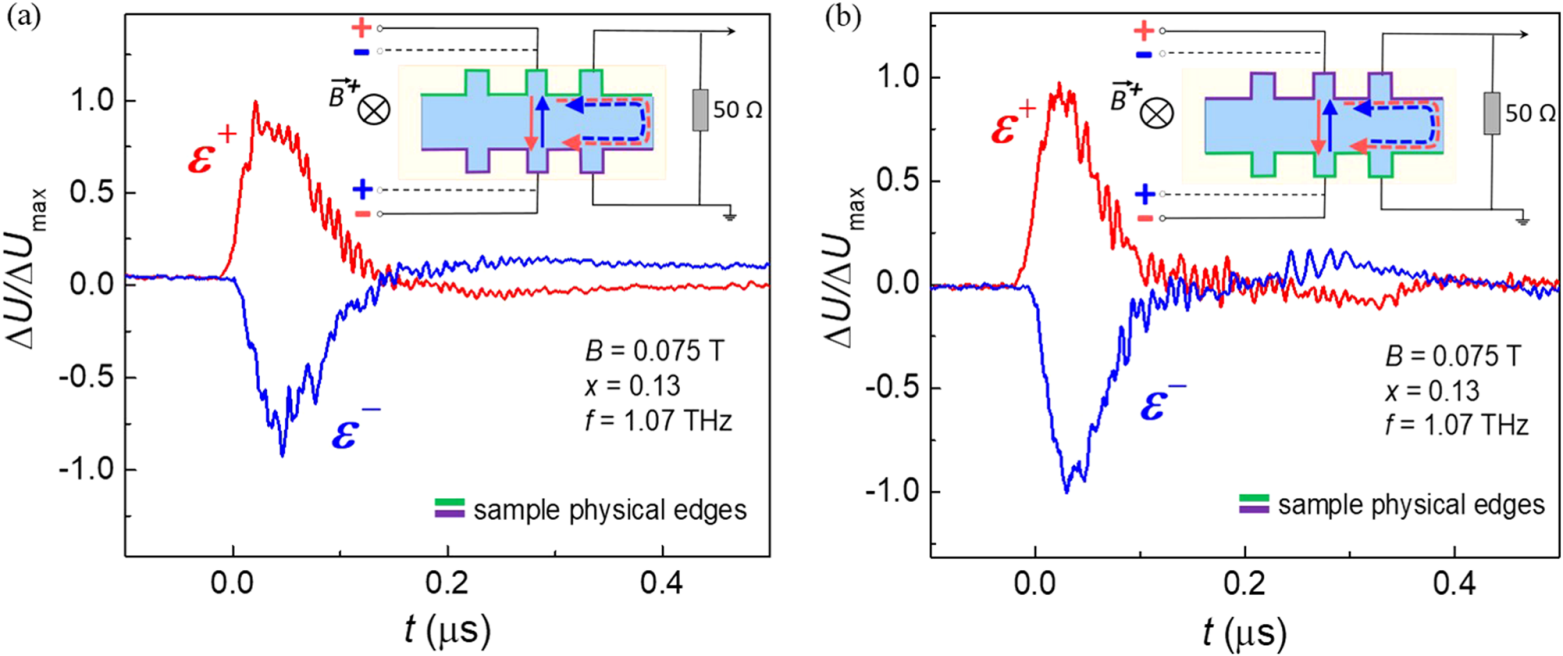
Nonlocal photoresponse before and after the sample rotation. Photoconductivity kinetics measured in the initial **(a)** and upside-down **(b)** sample positions. Physical sample edges are marked by respective colours in the insets. The film composition *x* = 0.13 corresponds to the topological phase, the magnetic field *B* +  = 0.075 T, the terahertz laser frequency is 1.07 THz.

A sample anisotropy could be such a factor. Then rotation of a sample by 180º around the magnetic field axis would change the photocurrent chirality for a given magnetic field direction and a bias polarity in the laboratory-linked coordinates. Such an experiment has been performed, and it turned out that this procedure does not change the photocurrent chirality (Fig. 6).

It means that the apparent experiment symmetry is broken by an external impact.

Non-homogeneous distribution of the radiation intensity over a sample could be such a factor. In particular, the photo-Nernst effect observed in graphene^30^ is due to the non-homogeneous sample heating by the incident radiation. On the other hand, no bias is needed for observation of the photo-Nernst effect. In our case, there is no photocurrent without a bias applied, and if the bias is applied, the photocurrent changes its sign depending on the bias polarity. Second, and most importantly, there are no noticeable changes in the effects observed upon moving the laser spot and, consequently, the possible thermal gradient along and across a sample (see the “Methods” section). For the photo-Nernst effect, such a dependence is observed, moreover, the photocurrent changes its sign upon moving the illumination spot from one sample edge to another. Stability with respect to the laser spot position observed in our case is most likely related to the fact that we operate with the incident laser power corresponding to the photoconductivity saturation, so that even relatively weak wings of the laser Gaussian spatial profile provide the same photoresponse. Such a photoconductivity saturation measured in the standard Hall bar experimental geometry in the zero magnetic field has been demonstrated in^31^.

The origin of an external factor providing appearance of the effect is still unclear. It is a matter of further studies.

## Conclusions

In summary, we have observed strong non-local terahertz photoconductivity in heterostructures based on thick Hg_1−*x*_Cd_*x*_Te films being in the topological phase. While the local conductivity in the structures studied is small, application of terahertz laser pulses results in appearance of edge photocurrents that exceed the dark signal by orders of magnitude. In particular, the photovoltage drop at the most remote couple of potential leads exceeds the respective dark voltage by more than 5 × 10^4^ when applying the terahertz power up to 10 kW. The edge photocurrents are chiral, i.e. they flow around a sample as long as the magnetic field and the electric bias are applied. The photocurrent chirality is reversed upon switching the magnetic field or the electric bias to the opposite one. The edge photocurrents reach maximum in small magnetic fields ~ 0.08 T, they drop down and disappear in higher fields. Appearance of the non-local photoconductivity is attributed to features of the interface between the topological film and the trivial buffer. It is suggested that the effect may be due to a macroscopic spin–orbit interaction.

## Methods

The samples under study were the same Hg_1−*x*_Cd_*x*_Te films as studied in^31–33^. The films were synthesized by MBE. ZnTe and CdTe buffer layers, a CdTe-rich mercury cadmium telluride relaxed layer, a 3D Hg_1−x_Cd_x_Te layer, and a CdTe-rich cap layer were successively grown on a GaAs (013) semi-insulating substrate (Fig. 1a). The active 3D Hg_1−*x*_Cd_*x*_Te layer thickness was about 4 µm. The solid solution composition *x* was controlled in situ by ellipsometry. The synthesis is described in detail in^25,26^.

The active layer composition was *x* = 0.13, 0.15 and 0.175. The first two films correspond to the topological phase, the third film belongs to the trivial phase (Fig. 1b). All films were of the *n*-type, with the electron concentration ~ 10^14^ cm^−3^ at the liquid helium temperature. More detailed sample characterization is presented in^32^.

The photoconductivity was excited by pulses of a gas NH_3_ laser of ~ 100 ns duration. The terahertz radiation frequency was 1.1, 2.0, or 3.3 THz with the power in a pulse up to 10 kW. The beam had an almost Gaussian profile with the spot size of 3–5 mm depending on the laser wavelength. The beam was centered at the middle of a sample. Within the experimental accuracy, moving the laser spot along and across a sample by 2–3 mm did not change the ettects observed. The photoconductivity measurements were performed by the 4-probe method for two opposite directions of the applied bias, as well as for the zero bias to control the amplitude of the photovoltaic contribution to the signal, if present. In our photoconductivity measurements, a 50 Ω resistor was connected in parallel to the potential leads to provide the impedance matching with the oscilloscope input (see Fig. 2). The equilibrium 2-probe resistance measured between these contact couples was by a factor of 5–10 higher, which means that the photocurrent between the respective contact couples was measured. More experimental details may be found in^34–36^.
